# Effectiveness of hybrid digital breast tomosynthesis/digital mammography in women presenting for routine screening at Maroondah BreastScreen, Australia: Interim analysis

**DOI:** 10.1016/j.breast.2025.104640

**Published:** 2025-10-27

**Authors:** M Luke Marinovich, Darren Lockie, Michelle Giles, Sally Doncovio, Georgina Marr, David Taylor, Tong Li, Brooke Nickel, Nehmat Houssami

**Affiliations:** a The Daffodil Centre, The University of Sydney, and Cancer Council NSW, Sydney, New South Wales, Australia; b Maroondah BreastScreen, Eastern Health, Victoria, Australia; c BreastScreen Victoria, Victoria, Australia; d Office of Research and Ethics, Eastern Health Institute, Eastern Health, Box Hill, Victoria, Australia; e Sydney School of Public Health, Faculty of Medicine and Health, The University of Sydney, Camperdown, New South Wales, Australia

## Abstract

Hybrid digital breast tomosynthesis (DBT)/digital mammography (DM) (mediolateral oblique from DBT with synthetic 2D, craniocaudal from DM) can potentially improve breast cancer detection and address longer DBT screen-reading time. This pre-specified interim analysis presents the first 5,000 (of target 20,000) hybrid DBT/DM screens in an ongoing prospective trial in BreastScreen Australia. Cancer detection rate for hybrid DBT/DM was 10.6/1,000 screens; recall rate was 4.2 %; median screen-reading time was 36 seconds; and mean glandular dose was 2.49 mGy. Cancer detection and recall rates for hybrid DBT/DM were consistent with DBT metrics in a previous pilot study; screen-reading time was approximately halved.

## Highlights

• Hybrid DBT/DM screening showed comparable breast cancer detection and recall to previous estimates for two-view DBT.
• Screen-reading time was approximately halved.
• Interim results are encouraging for the potential feasibility of hybrid DBT/DM screening.

## 1 Background

Evidence from prospective trials and retrospective studies has shown that population breast cancer screening using digital breast tomosynthesis (DBT) with (or instead of) standard digital mammography (DM) improves screening detection measures compared to DM alone, but results vary across screening settings. [1,2] In the Australian screening program (BreastScreen), DBT is not used for routine screening because it is unclear whether the enhanced cancer detection observed in international studies will transfer to the Australian setting, and if additional detection has incremental benefit to screening with DM alone. A pilot trial undertaken in BreastScreen Victoria showed that DBT increased cancer detection as well as recall rates; in addition, substantially longer screen-reading times for DBT relative to DM were reported. [3]

A hybrid DBT/DM screening approach, utilising the mediolateral oblique (MLO) view from DBT (with synthetic 2D from DBT acquisition) and the craniocaudal view from DM, has the potential to enhance cancer detection while addressing logistical and resourcing challenges associated with the longer screen-reading time for DBT. This trial (in progress) aims to evaluate the effectiveness of hybrid DBT/DM screening through a comparison with standard DM in women presenting for routine breast screening at Maroondah BreastScreen, Eastern Health in Victoria, Australia. [4] In the present interim analysis, we report initial screening outcomes, along with reading time and radiation dose, for the hybrid DBT/DM arm to describe the feasibility and safety of this approach.

## 2 Methods

Detailed study methods of this prospective, non-randomised trial, including this prespecified interim analysis, are described in the published trial protocol. [4] The study was approved by the Eastern Health HREC (LR23-059-102540), and is registered with the Australian New Zealand Clinical Trials Registry (ACTRN12623001144606, https://www.anzctr.org.au/). The first 5,000 women enrolled in the hybrid DBT/DM arm (of the target sample size of 20,000 in the intervention group) at Maroondah BreastScreen are included in this analysis. The primary outcomes of cancer detection rate (CDR; per 1,000 screens) and recall rate (percent) were derived with exact (Clopper-Pearson) binomial 95 % confidence intervals (CIs). Secondary outcomes of hybrid DBT/DM reading time per reader (seconds) and glandular radiation dose (milligray, mGy) per screening examination are described with the median (interquartile range, IQR) and mean (standard deviation, SD), respectively. Comparison with the DM screening arm has not been undertaken, in accordance with the prespecified analysis which was undertaken to monitor the hybrid intervention. Because BreastScreen has ongoing monitoring of screening metrics against national standards for quality assurance, we did not set a pre-specified stopping rule for the trial and only planned to report the interim results for hybrid DBT/DM to BreastScreen Victoria's research governance committee (who have endorsed trial continuation). For context and interpretation of the interim findings, estimates from DBT and DM results from the earlier pilot trial [3] (collected at the same study site as hybrid DBT/DM) are provided.

## 3 Results

Table 1 presents baseline characteristics, and Table 2 summarises prespecified interim primary and secondary outcomes for hybrid DBT/DM; results from the previous pilot trial are also shown.

**Table 1:** Baseline characteristics for hybrid DBT/DM with comparisons to pilot trial undertaken in same screening service.

Characteristic	Hybrid DBT/DM	Pilot trial [3]: Two-view DBT	Pilot trial [3]: DM
Number of screens	5,000	5,018	5,166
Age (years)			
Mean (SD)	59.1 (8.6)	58.0 (8.4)	62.3 (8.1)
Range	40–90	40–91	40–93
Screening round			
First (prevalent) screen	798 (16.0 %)	980 (19.5 %)	350 (6.8 %)
Subsequent (incident) screen	4,202 (84.0 %)	4,038 (80.5 %)	4,816 (93.2 %)
Breast symptom reported	385 (7.7 %)	380 (7.6 %)	283 (5.5 %)
Family history of breast cancer	1,682 (33.6 %)	1,492 (29.7 %)	1,499 (29.0 %)
Personal history of breast cancer	80 (1.6 %)	33 (0.7 %)	31 (0.6 %)

**Table 2:** Interim monitoring results for hybrid DBT/DM with comparisons to pilot trial undertaken in same screening service.

	Hybrid DBT/DM		Pilot trial [3]: Two-view DBT		Pilot trial [3]: DM	
	n	Estimate	n	Estimate	n	Estimate
**Number of screens**	5,000	-	5,018	-	5,166	-
**Primary outcomes**						
CDR, per 1,000 (95 % CI)	53	10.6 (7.9–13.8)^a^	49	9.8 (7.2–13.0)	34	6.6 (4.6–9.2)
Recall rate, % (95 % CI)	209	4.2 (3.6–4.8)	210	4.2 (3.6–4.8)	155	3.0 (2.6–3.5)
**Secondary outcomes**						
Median reading time per reader, seconds (IQR)^b^	-	36 (23–60)	-	67 (46–105)	-	16 (10–29)
Mean glandular dose, mGy (SD)^c^	-	2.49 (0.75)	-	2.55 (0.83)	-	1.32 (0.42)

Abbreviations: CDR, cancer detection rate; CI, confidence interval; DBT, digital breast tomosynthesis; DM, digital mammography; IQR, inter-quartile range; mGy, milligray; SD, standard deviation.

a One recalled participant without assessment outcome assumed to be false positive recall. If true positive recall assumed, CDR = 10.8 per 1,000 (8.1–14.1).

b First two reads only (10,000 reads total for hybrid DBT/DM). As per methodology adopted in the pilot trial, [3] values greater than 300 seconds were deemed implausible (probably attributable to the reader not closing read or interrupted reading) and have been truncated.

c Based on hybrid DBT/DM radiation data available for 4,888 screens.

### 3.1 Cancer detection rate

The CDR for hybrid DBT/DM screening was 10.6/1,000 screens (95 % CI: 7.9–13.8). This estimate assumes false-positive recall for one participant who was recalled but did not attend for further assessment. If true positive recall is assumed, the CDR for hybrid DBT/DM is 10.8/1000 screens (95 % CI: 8.1–14.1). These CDR estimates are consistent with the estimate for (two-view) DBT from the pilot trial, and qualitatively larger than the CDR for DM (Table1).

### 3.2 Recall rate

The recall rate for hybrid DBT/DM screening was 4.2 % (95 % CI: 3.6–4.8), similar to the recall rate for DBT observed in the pilot trial (and larger than the recall rate for DM).

### 3.3 Screen-reading time

The median screen-reading time for hybrid DBT/DM per reader was 36 seconds (IQR: 23–60). This estimate is lower than the median screen-reading time for DBT, but greater than the median estimate for DM, from the pilot trial.

### 3.4 Radiation dose

The mean glandular dose per examination for hybrid DBT was 2.49 mGy (SD: 0.75). This is consistent with estimate for DBT screening from the pilot trial, and higher than the mean glandular dose for DM (Table 1).

## 4 Discussion

This interim analysis of the first 5,000 hybrid DBT/DM screening examinations found results for the primary outcomes that were consistent with estimates for two-view DBT observed in the earlier pilot trial undertaken in the same screening service. [3] The CDR for hybrid DBT/DM (10.6/1,000) was slightly larger than for DBT (9.8/1,000), but estimates are imprecise due to the relatively small sample sizes (noting the trial's target accrual of 20,000 into each group will achieve greater precision). The recall rate estimate for hybrid DBT/DM was the same as that observed in the DBT pilot (4.2 %). These interim results extend international findings of improved cancer detection from the DBT MLO view relative to DM, [5] and provide reassurance that the enhanced cancer detection by two-view DBT observed in the Australian pilot trial is maintained using hybrid DBT/DM without an unexpected additional increase in recall.

The interim analysis also suggests that hybrid DBT/DM is likely to result in a reduction in screen-reading time relative to DBT (acquired in both views), with an approximate halving in median reading time. Although median reading time was still longer than that for DM in the pilot, this result (if maintained in the final analysis) is encouraging for the potential feasibility of hybrid DBT/DM screening. Estimates of radiation dose show similar mean glandular doses for hybrid DBT/DM and DBT; radiation dose will be further explored in the final analysis.

This ongoing prospective trial represents the first Australian study of DBT that is sufficiently powered to demonstrate improvements in cancer detection relative to DM, and the first trial internationally of a hybrid DBT/DM approach. Interim results suggest that hybrid DBT/DM is likely to replicate the enhanced cancer detection shown for full-acquisition DBT with similar recall, but with lower screen-reading time. Direct comparison to screening metrics for DM, including stratification by age-group and breast density, will be presented in the final trial report. Participant recruitment is ongoing and is expected to be completed in mid-2026.

## CRediT authorship contribution statement

**M Luke Marinovich:** Formal analysis, Methodology, Writing – original draft, Writing – review & editing. **Darren Lockie:** Investigation, Methodology, Writing – review & editing. **Michelle Giles:** Methodology, Project administration, Writing – review & editing. **Sally Doncovio:** Methodology, Project administration, Writing – review & editing. **Georgina Marr:** Data curation, Resources, Writing – review & editing. **David Taylor:** Methodology, Writing – review & editing. **Tong Li:** Methodology, Writing – review & editing. **Brooke Nickel:** Methodology, Writing – review & editing. **Nehmat Houssami:** Conceptualization, Funding acquisition, Methodology, Writing – original draft, Writing – review & editing.

## Ethics approval and consent to participate

The study has been approved by the Eastern Health HREC (LR23-059-102540).

## Consent for publication

Not applicable.

## Availability of data and materials

The datasets generated and/or analysed during the current study are not publicly available due to data confidentiality agreements with data custodians.

## Sources of Funding

This work is funded by a 10.13039/501100001026National Breast Cancer Foundation (NBCF) Endowed Chair Grant (EC-21-001; 2021-31), and co-funded by a 10.13039/501100000925National Health and Medical Research Council (NHMRC) Investigator/Leader Grant (1194410; 2021-25), both awarded to NH. The funders had no role in the study design; in the collection, analysis and interpretation of data; in the writing of the manuscript; and in the decision to submit the manuscript for publication.

## Declaration of competing interest

Given his role as Editorial Board Member, M Luke Marinovich had no involvement in the peer-review of this article and has no access to information regarding its peer review. The other authors declare that they have no competing interests.
